# Early Life Interventions Can Shape Aging

**DOI:** 10.3389/fendo.2022.797581

**Published:** 2022-02-25

**Authors:** Andrzej Bartke, Liou Y. Sun, Xinna Li, Richard A. Miller

**Affiliations:** ^1^Department of Internal Medicine, Southern Illinois University School of Medicine, Springfield, IL, United States; ^2^Department of Biology, University of Alabama at Birmingham, Birmingham, AL, United States; ^3^Department of Pathology and Paul Glenn Center for Biology of Aging Research, University of Michigan, Ann Arbor, MI, United States

**Keywords:** Developmental Origins of Health and Disease (DOHaD), postnatal development, early life interventions, growth hormone, mutant mice, aging, healthspan, lifespan

## Abstract

It is well documented that the environment of the developing fetus, including availability of nutrients and presence of toxins, can have major impact on adult phenotype, age-related traits and risk of chronic disease. There is also accumulating evidence that postnatal environment can impact adult characteristics related to evolutionary fitness, health, and aging. To determine whether early life hormonal interventions can alter trajectory of aging, we have examined the effects of early life growth hormone (GH) replacement therapy in Prop1^df^ (Ames dwarf) mice which are GH deficient and remarkably long lived. Twice-daily GH injections between the ages of two and eight weeks completely normalized (“rescued”) a number of adult metabolic characteristics believed to contribute to extended longevity of these mutants. Importantly, longevity of Ames dwarf mice was reduced by early life GH treatment. This was associated with histone H3 modifications. We conclude that the trajectory of mammalian aging can be modified by early life interventions. Mechanistic links among interventions during postnatal development, adult metabolic characteristics, aging, and longevity, apparently involve epigenetic phenomena.

## Introduction

### Developmental Origins of Health and Disease

Impact of early life events on the characteristics of an adult organism is a concept which is well established in developmental biology. Studies dating back to the middle of the last century documented the potential of environmental changes during development to produce alterations in adult phenotype that mimic the effects of genetic mutations, the so-called “phenocopies.” Early interpretation of experimental evidence in this field, including proposal of the concept of canalization of development (1), represent classical milestones in the history of developmental biology. Studies of people born in The Netherlands during or after the period of extreme food shortages toward the end of World War II (the Dutch hunger winter) led to renewed interest of biologists, clinicians, and demographers in developmental programming of adult characteristics. These studies provided substantial evidence that malnutrition or starvation during pregnancy can increase the risk of metabolic disturbances and chronic disease in the offspring, including hypertension, cardiovascular disease, and diabetes (2, 3). Subsequent studies in other populations experiencing periods of severe food deprivation (4–6) and in experimental animals, primarily sheep and rats subjected to calorie or protein restriction during pregnancy (7–9), firmly established the concept of Developmental Origins of Health and Disease (DOHaD). In addition to documenting the impacts of maternal nutrition, studies in this field provided evidence that exposure to toxic compounds [including endocrine disrupting chemicals (EDCs)] or to chronic stress during pregnancy can have significant negative effects on the health of the offspring, including disturbances of glucose homeostasis, obesity, and cardiovascular disease (10–12). Intriguingly, some of these effects can be transgenerational, influencing the offspring of the prenatally exposed individuals (13, 14). The mechanisms of early life events on adult functioning and disease risk encompassed in the concept of DOHaD are unlikely to include changes in the DNA sequence or integrity (mutations, deletions, etc.), and have been linked to epigenetic effects, primarily changes in the methylation and/or acetylation of DNA and chromosomal proteins leading to alterations in gene expression (15, 16).

Most of the studies in this area did not specifically address the potential impact of intrauterine environment and prenatal exposure to noxious factors on aging and longevity. However, metabolic dysregulation and increased risk of chronic disease, which stood out as characteristics of the affected individuals, also represent some of the most consistent hallmarks of aging. Thus, it seems reasonable to suggest that the effects described in many DOHaD studies are either associated with or are, indeed, due to induction of accelerated and/or premature aging. In support of this interpretation, month of birth (which represents a proxy characteristic for environmental effects) was shown to influence the chances of survival to the age of 100 years (17), and meta-analysis of available datasets linked lifespan to prenatal diet manipulations (18). In the context of the ongoing SARS-CoV-2 pandemic, it is interesting to point out that maternal exposure to the 1918 influenza pandemic was shown to increase the risk of cardiovascular disease in the offspring (19). Indeed, it has been suggested that both maternal infections with SARS-CoV-2 and the stress caused by the pandemic are likely to have detrimental effects on the health and aging of the 2021/2022 birth cohorts (20).

### Effects of Post-Natal Conditions and Interventions

Our laboratory is interested in the impact of post- (rather than pre-) natal environment and interventions during this period on aging and age-related disease (21–23). Although the impact of postnatal events on programming the trajectory of aging received relatively little attention in previous studies, there are indications that this period of development can shape adult characteristics related to health and aging. Postnatal overfeeding was reported to alter numerous aspects of metabolic regulation in rats (24), peripubertal growth hormone (GH) treatment reversed radiation-resistance phenotype and cancer resistance in Lewis rats (25), and neonatal ghrelin action was shown to program development of hypothalamic feeding circuits and influence adult metabolism in mice (26). In humans, exposure to low ambient temperatures during early childhood was shown to promote brown adipose tissue (BAT) thermogenesis in adulthood, indicating that childhood represents a sensitive period in BAT plasticity (27),; childhood hunger episodes were reported to be associated with health deficits and faster aging, with the difference in health deficits between hungry and non-hungry individuals increasing with age, implying that children who suffered from hunger age faster (28). Indeed, early life adversities as well as social disadvantage were reported to affect epigenetic age acceleration and adult adiposity, respectively (29, 30). Relevant to the issue of healthy aging, rural living in early life was recently reported to be an independent risk factor for lower levels of cognitive functioning in later life (31). Intriguingly, both external and endocrine environment experienced by juveniles impact adult and late life characteristics also in wild animals living in their natural environment. In female bighorn sheep, population density and environmental temperature during the first year of life influenced fitness in terms of old age reproductive success and survival, even though no changes in the rate of senescence were detected (32). In wild spotted hyenas, endocrine characteristics of juveniles were shown to predict their life history trade-offs and longevity (33). In Seychelles warblers, reduced food availability in early life delayed both the onset of breeding and senescence (34). In terms translational potential of laboratory research findings into interventions to reduce risk of chronic disease and promote healthy aging in humans, we believe that the peripubertal period might represent a practical “time window” when an intervention can impact aging. Targeting the interventions to this period could benefit from greater accessibility and lower risk of adverse side-effects when compared to the periods of intrauterine development or early infancy.

### Early Life Endocrine Intervention in Long-Lived Mice

In a “proof-of-concept” study, we decided to determine whether aging-related characteristics and longevity of an exceptionally long-lived mouse mutant can be normalized (“rescued”) by hormonal intervention limited to a brief period of rapid peripubertal growth. For these studies we have used Ames dwarf mice, animals which are homozygous for a loss-of-function mutation of the Prop-1 gene which controls differentiation of several types of hormone-producing cells in the anterior pituitary (35, 36). The resulting defects in endocrine function lead to slower postnatal growth, delay of puberty, and severe reduction of adult body size, as well as a major extension of longevity (35–37). Importantly, delayed and slower aging and extended longevity of these animals are associated with various indices of healthy aging and extension of the healthspan, a period of life free of disease, frailty, and functional impairments (35, 38). Overlap of the phenotypic characteristics of these, and other GH-related, long-lived mouse mutants, with those of GH-deficient and GH-resistant humans was discussed in our recent publications (39, 40). The endocrine intervention employed in our studies consisted of twice-daily injections of growth hormone (GH), one of the hormones that these animals fail to produce and was limited to a period of six weeks starting at the age of one or two weeks, when mice are still suckling (21, 22).

As expected, GH injections stimulated somatic growth of juvenile Ames dwarf mice.

**Figure 1:**
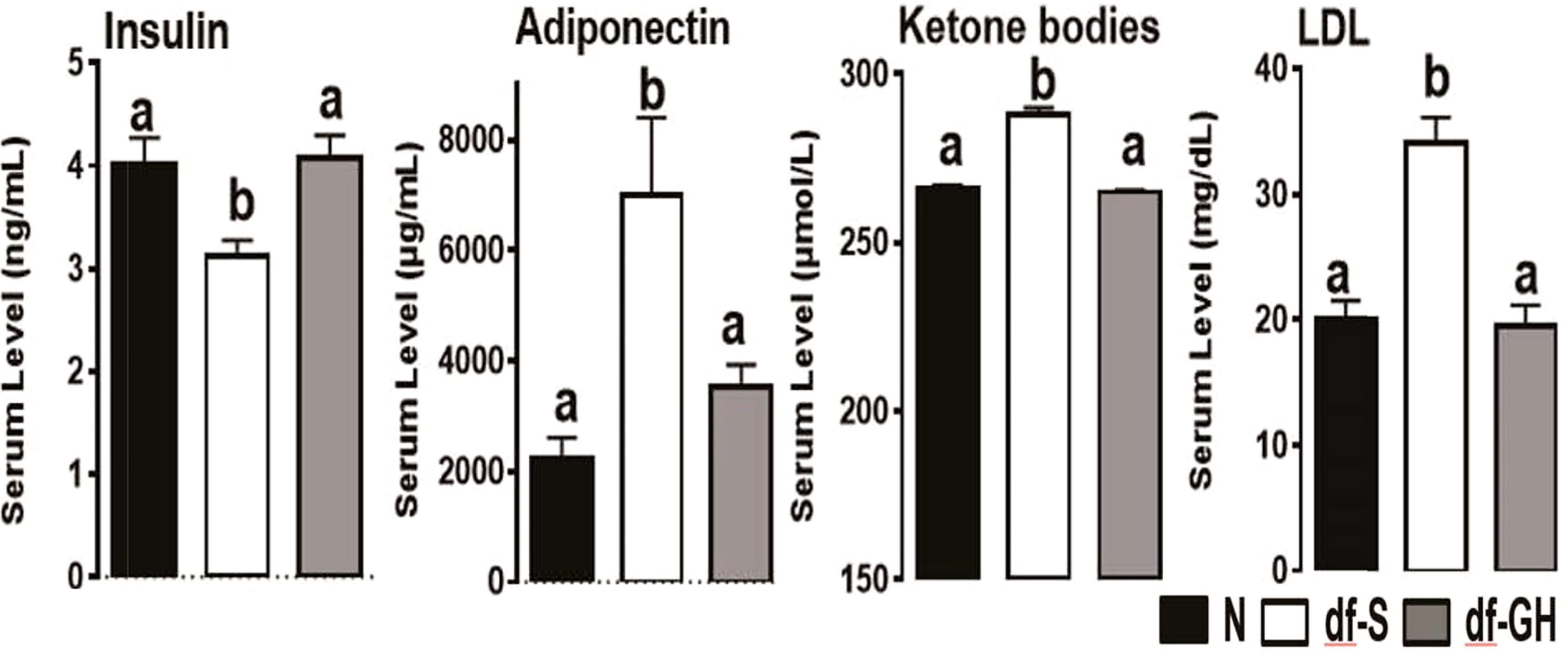
Metabolic alterations in responses to early GH treatment. Various plasma parameters from male Ames dwarf (*Prop1*^df^) and Littermate control male mice (N) subjected to early-life GH treatment. Saline-treated-control mice (black bar), Saline-treated-dwarf mice (white bar), and GH-treated-dwarf mice (grey bar), ^a,b^ values that do not share a superscript letter are significantly different (p < 0.05). Data represent the means ± SEM (19).

**Figure 2:**
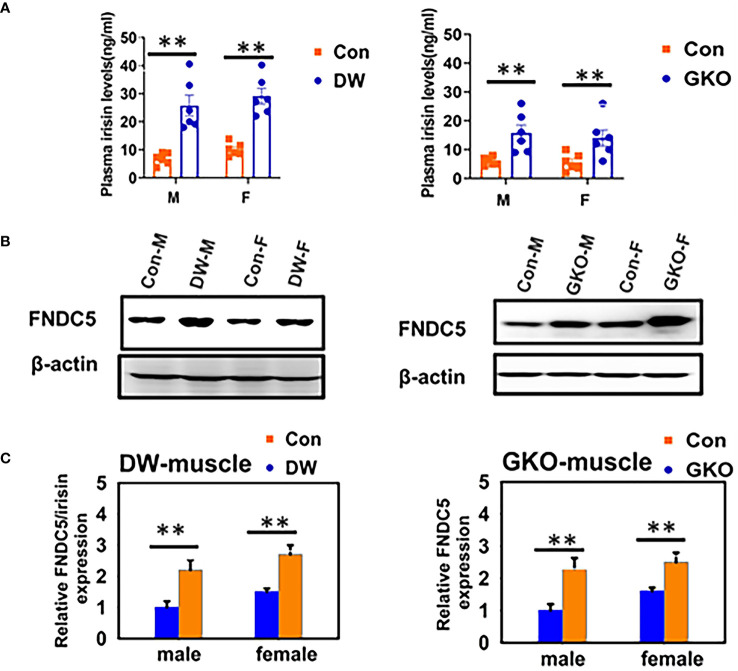
Plasma irisin levels and expression of FNDC5 in muscle tissue of two kinds of slow aging mice, Snell DW, and GHRKO. **(A)** Irisin content was measured by ELISA on plasma samples of 24-week-old WT and mutant mice (DW, GHRKO, abbreviated GKO). Data are shown as mean ± SEM for each group (n = 6). **P < 0.001 versus WT. **(B)** Cell lysate was prepared from gastrocnemius muscle of 24-week-old WT and slow aging mice, and protein levels of *FNDC5* were measured by western blotting. Representative gel images are shown. **(C)** Relative protein expression was normalized to *β-actin* levels. Values are mean ± SEM (n = 4). **P < 0.05 versus WT (38).

Growth curve (a plot of body weights, determined daily) of GH-injected dwarfs was much steeper than the growth curve of vehicle-injected (control) dwarfs, with a slope closely resembling the growth curve of their normal (wild type, WT) siblings. When the injections were stopped, the dwarf mice quickly reverted to minimal daily gains in body weight which are typical of untreated or vehicle-injected dwarfs. Consequently, adult body weight of dwarfs that had been treated with GH during early life was intermediate between the values measured in normal (WT) mice and in control (vehicle-injected) dwarfs. The impact of early life GH treatment on adult phenotype, and specifically on traits related to aging, was substantial, and supported our hypothesis concerning impact of early life intervention on adult characteristics. Many characteristics of GH-treated Ames dwarf mice, measured one year or later after the GH therapy was finished, were completely rescued, that is no longer differed from the same characteristics of normal animals. Growth hormone treatment reduced the levels of adiponectin, low density lipoprotein, and ketone bodies, and increased the levels of insulin (**Figure 1**). Moreover, this intervention reduced metabolic rate (oxygen consumption [VO2] per g body weight) and increased respiratory quotient, completely eliminated protection from age-related astrogliosis, and completely or partially normalized a number of inflammatory markers in the liver and epididymal white adipose tissue, as well as hepatic expression levels of a variety of genes related to stress responsive pathways (21, 22, 41). Subsequent collaborative studies identified other adult characteristics related to mechanisms of aging that were substantially altered in Ames dwarf mice and largely or completely normalized by six weeks of treatment with GH in early life. These characteristics include hepatic production of hydrogen sulfide, a compound recently shown to be importantly involved in mediating the effects of calorie restriction (42), and muscle levels of FNDC5 protein, a parent molecule of irisin, one of the factors that regulate insulin sensitivity (43). In a previous work (43), we have noted changes in thermogenesis, macrophage balance towards anti-inflammatory states, and diminished cytokine mRNA production in both subcutaneous and intra-abdominal fat depots of Snell dwarf (Pit-1^dw^) and GHRKO mice. Curiously, these changes were not seen in mice where GHR had been disrupted in only fat tissue, suggesting that they represented an indirect effect of GH signals in another tissue. Studies of muscle-specific GHRKO mice showed that disruption of GHR in muscle was sufficient to reproduce nearly all of the changes noted in fat tissue of mice with a global deletion of GHR. Muscle effects on fat are thought to reflect, in part, release from muscle of irisin, a product of FNDC5 protein. Indeed, plasma irisin levels were found to be elevated in Snell and GHRKO mice, in parallel to elevation of muscle FNDC5 levels [**Figure 2**; excerpted from (43)]. It will be of great interest to see if early-life manipulation of GH signals, or of adult-life induction of GH deletion, can lead to parallel changes in fat, muscle, irisin and inflammatory macrophage status. Importantly, GH therapy during early life reduced longevity of Ames dwarf mice (21, 22).

## Discussion

### Conclusions, Interpretation, and Future Directions

When compared to their normal (WT) siblings, dwarf mice represent clear features of slow pace-of-life: reduced growth rate, delayed puberty, and reduced fecundity (35, 37, 38). We suspect that the impact of early life GH therapy on longevity detected in our studies was due primarily to GH stimulation of anabolism, growth, and maturation, that is actions that represent acceleration of the pace-of-life. There is increasing appreciation that longevity of an organism is determined by a balance of various trade-offs and regulatory loops, and that association of the pace-of-life with the rate of aging figures prominently among these relationships. However, other actions of GH also could have been involved in partially rescuing (that is shortening) their longevity. Growth hormone has well documented effects on insulin signaling (44), immune function (45), stress resistance (46, 47), and DNA repair (25), and each of these factors has important role in the regulation of aging and longevity (48). Our recent findings concerning histone modifications in Ames dwarf mice treated with GH (49) suggest that epigenetic mechanisms might have been involved in mediating the effects of this early life intervention on adult characteristics and longevity.

Resembling the effects of a short period of GH replacement therapy on adult body weight, longevity of dwarf mice was not completely normalized (rescued). Growth hormone-treated dwarfs lived shorter than dwarfs injected with vehicle, but still longer than normal (WT) mice. As indicated earlier in this section, early life GH treatment completely normalized many, but not all, characteristics related to aging. Presumably, longevity of GH-treated Ames dwarf mice is influenced by parameters that were not fully normalized (and/or not examined in the present study). Nevertheless, our findings demonstrate than intervention limited to several weeks before and around the time of sexual maturation can alter aging and life expectancy.

We realize, of course, that without additional evidence, the effects of our studies of hormone replacement therapy in a mutant with severe endocrine deficiencies (21–23) cannot be assumed to represent normal regulatory mechanisms in wild type (WT, that is “genetically normal”) animals. Nevertheless, we are tempted to speculate that targeting hormonal, pharmacological, nutritional, or environmental interventions to this particular stage of postnatal development is likely to influence the trajectory of aging in normal mice. We are particularly interested in the possibility of identifying interventions which could be applied during adolescence to promote extension of the healthspan and healthy aging, possibly also leading to extension of longevity. We suspect that interventions capable of affecting the energy metabolism to slow the pace-of-life may be particularly promising in this regard. We are currently testing these possibilities utilizing mild alterations in environmental (ambient) temperature and drugs developed for the treatment of diabetes.

## Author Contributions

Concept: AB. Writing: AB, LS, XL, and RM. Funding: AB and RM. All authors contributed to the article and approved the submitted version.

## Funding

Writing of this article and our recent and current studies of this topic were supported by American Diabetes Association grant ADA 1–19-IBS-126 and NIA R21-AG062985 (AB). Work in the Miller lab was supported by NIA grants P30-AG024824 and the Glenn Foundation for Medical Research.

## Conflict of Interest

The authors declare that the research was conducted in the absence of any commercial or financial relationships that could be construed as a potential conflict of interest.

## Publisher’s Note

All claims expressed in this article are solely those of the authors and do not necessarily represent those of their affiliated organizations, or those of the publisher, the editors and the reviewers. Any product that may be evaluated in this article, or claim that may be made by its manufacturer, is not guaranteed or endorsed by the publisher.
